# The effective and safe method for preventing and treating bacteria-induced dental diseases by herbal plants and a recombinant peptide

**DOI:** 10.4317/jced.55717

**Published:** 2020-06-01

**Authors:** Abbas Tanhaieian, Solmaz Pourgonabadi, Majid Akbari, Hamideh-Sadat Mohammadipour

**Affiliations:** 1 Dental research center, School of Dentistry, Mashhad University of Medical Sciences Mashhad, Iran; 2 Oral and maxillofacial department, School of Dentistry, Mashhad University of Medical Sciences, Mashhad, Iran; 3 Department of Restorative and Cosmetic Dentistry, School of Dentistry, Mashhad University of Medical Sciences, Mashhad, Iran; 4 Dental materials research center, School of Dentistry, Mashhad University of Medical Sciences, Mashhad, Iran

## Abstract

**Background:**

This study was conducted aimed at evaluating the antibacterial property of the recombinant peptide of bacteriocin entrocin P (EnP), the essential oil of Cuminum cyminum, and the extract of *Ferulago angulata* on some oral pathogens. Besides, the cytotoxicity of EnP was evaluated.

**Material and Methods:**

The antimicrobial property was tested on *streptococcus mutans* (ATCC 35668), *streptococcus salivarius* (ATCC 9222), *streptococcus oralis* (ATCC 35037), and *Enterococcus faecalis* (ATCC 29212), using the microbroth dilution method. The 0.2% Chlorhexidin (CHX) mouthwash was used as the control group. Besides, the cytotoxicity analysis was done on gingival fibroblasts by the MTT colorimetric method. The data were reported using descriptive methods, and analyzed by one-way ANOVA, and Tukey’s HSD test.

**Results:**

The strongest bacteriostatic and bactericidal effects of *C. cyminum* and *F. angulata* were observed for *S.mutans* and *S. oralis*, respectively (with the MIC and MBC value being 62.5 μg/mL). The antibacterial properties of EnP were comparable to those of CHX, being several times stronger than medicinal plants (1-14 μg/mL). Based on the cytotoxicity evaluation, there was no statistically significant difference observed between the cytotoxicity of the control group and that of Enp for three evaluations, except after 72 hours when the cell viability at the concentration of 3.75 µg/ml was significantly lower than that of the control group (*P*=0.05). However, no concentration of EnP was observed to be over 50% of the growth inhibition (IC50) of the fibroblasts for the three evaluations.

**Conclusions:**

EnP could be utilized in dental materials as a natural and safe antimicrobial agent against oral streptococci and E. faecalis, being as effective as CHX mouthwash.

** Key words:**Antimicrobial peptide, Bacteriocin Entrocin P, Chlorhexidine, Cuminum cyminum, Enterococcus faecalis, Ferulago angulata.

## Introduction

Dental caries is a prevalent disease that severely affects the patient’s quality of life due to producing an acute pain, as well as the mastication ability, and aesthetic aspects. Besides, it undermines the patient’s self-estimate as it leads to tooth extraction (1). Thus, many attempts have been made to apply new materials and methods so as to decrease the prevalence of this disease (2).

Dental caries is closely related to the presence of oral biofilms on the tooth surface. One of the most complex biofilm structures in nature is the human dental plaque that causes a variety of oral infections, including dental caries, pulp and periodontal diseases (3). Oral biofilms consist of dental plaque and bacteria such as *Streptococcus mutans* (*S. mutans*), which are capable of sticking to the tooth, proliferate, and produce acidic byproducts. The produced lactic acid can dissolve the mineralized components of dental enamel and dentin, resulting in caries formation (1). The role of *S. mutans* as one of the most important factors leading to caries formation has been recognized. Since the Viridans Streptococci group can lead to more oral diseases, including bacterial endocarditis, respiratory diseases, streptococcal septicemia, meningitides, and other bacterial diseases, the use of antimicrobial agents in the chemical elimination of the microbial dental plaque is not only a worthy solution for caries prevention, but it also controls such systemic diseases (2).

Being regarded as a broad-spectrum antimicrobial agent, Chlorhexidin (CHX) has bacteriostatic properties at low concentrations and bactericidal effects at high concentrations, resulting in the coagulation and precipitation of bacterial cytoplasm. The antimicrobial effects of CHX have been attributed to cationic bisbiguanide molecules. The major advantages of chlorhexidine compared with other antimicrobial agents such as sodium hypochlorite include the lower cytotoxicity and the lack of any unpleasant smell and taste. However, it has some disadvantages, including its inability to dissolve organic substances, necrotic tissues and remove the smear layer. Although the effect of CHX has been verified on *S. mutans*, it has some side effects on the tooth and oral mucosa that limit the long-term usage of this mouthwash; tooth discoloration (as the most common consumer complaint), taste changes, supragingival calculus increases, allergies, and oral lesions are from among the most common side effects of CHX (4,5).

The application of antimicrobial agents is not limited to the caries prevention and treatment. The elimination of microorganisms from the root canal is crucial for the successful endodontic treatment, emphasizing the need for chemical irrigation in addition to the mechanical preparations (6). The most common pathogen that induces endodontic failures is titled *Enterococcus faecalis*. Sodium hypochlorite (NaOCl) and CHX in both gel forms and liquid solutions are proposed to eliminate such microorganisms from the space of the root canal and dentinal tubules. However, both of these agents have some disadvantages. Sodium hypochlorite has an unpleasant taste, high toxicity, leaves stains, corrodes instruments, and burns the surrounding tissues. In addition, it cannot remove the smear layer but reduces the elastic modulus and flexural property of dentin (7).

Considering the increase in the bacterial resistance to antibiotics and the shortcomings of chemical agents, it seems logical to look for other effective alternatives with fewer side effects. In recent years, the use of natural products has attracted a lot of attention in the preventive dentistry, since it looks to be promising in reducing the side effects of chemical antimicrobial agents.

Antimicrobial peptides (AMP) have been recently acknowledged as potential alternatives to traditional antimicrobial agents and antibiotics due to their ability to specifically target bacterial biofilms, thereby leading to the prevention of biofilm formation and dissolution of the existing biofilms (3). They are generally composed of 12–50 amino acids with a net positive charge, being produced by many sources, including bacteria, insects, and mammals (8).

The pediocin-like bacteriocin, termed enterocin P (EntP), is considered to be of broad-spectrum antimicrobial properties against the spoilage bacteria such as *E. faecalis*, and other food-borne pathogens (9). EntP exhibits antimicrobial effects by making specific holes in the cytoplasmic membranes of bacteria. These potassium ion-conducting pores impair the electrochemical transmembrane potential through the accumulation of potassium ions inside the cells (10).

In recent years, medicinal plants have gained a considerable amount of attention as natural components serving antimicrobial and free radical scavenging activities, due to their safety and recognition by consumers. They can defend against microorganisms and prevent a wide range of diseases such as cancers, cardiovascular and neurodegenerative diseases (11).

*Cuminum cyminum*, also known as cumin or Jeera (Zeera, in Persian), is an aromatic plant from the Apiaceae family, used as food flavors, added to fragrances, and utilized in medical preparations (12). Cumin possesses numerous medicinal properties. It is used in the treatment of mild digestive disorders, bronchopulmonary disorders, as a cough remedy, and as an analgesic (13). The antibacterial property of this plant has been established in previous studies (14,15).

*Ferulago angulata* is a perennial member of the Apiaceae family that grows in the western mountainous regions of Iran. The biologic properties of this plant are attributed to the essential oils present in all parts of the plant, including its stems, leaves, flowers, and seeds (16). Ferulago species are used for their flavoring agents as well as their sedative, tonic, digestive, and anti-parasitic properties (17). The toxicity and mutagenicity of *F. angulata* extract (18-21) and *C. Cyminum* (22,23) were evaluated on several human cells in both *in vitro* and *in vivo* experiments, with the biocompatibility of them confirmed.

Considering the potential antimicrobial properties of herbals and EnP to substitute chemical antimicrobial agents, this study was conducted aimed at investigating the antimicrobial effects of the EnP peptide, *C. cyminum*, and *F. angulata* extracts, using the evaluation methods of the routine bacterial growth inhibition assay, the minimum inhibitory concentration assay (MIC), and the minimum bactericidal concentration (MBC) in regard to several bacteria involved in dental caries and endodontic infections. Besides, the cytotoxicity property of EnP was assessed on human gingival fibroblast cells.

## Material and Methods

-The preparation of the plant extract and essential oils

Different parts of *C. cyminum* and *F. angulata* from Apiaceous family were collected from the plants cultivated at the Center of Medicinal Plants Research of Mashhad, Khorasan Razavi Province, Iran, in 2018. The herbarium of the plant was verified by the botany department of Ferdowsi University, Mashhad, Iran. All parts of the plant containing seed and leaves were rinsed with distilled water and then dried at room temperature in a dark and cold place, for a week. To prepare the herbal essence and extracts, all parts of the plants were used and ground into a fine powder.

The herbal essence of Cumin was extracted from 100 grams of the crushed herbal parts, using the Clevenger apparatus for 6 hours. After isolation, the essence obtained was dried over anhydrous sodium sulfate and kept in a dark vial, before use.

To prepare the herbal extract of Chevile, the plant powder was after drying added to an Erlenmeyer flask containing 80% ethanol and distilled water, with the powder to solvent ratio of 1 to 6. To extract the effective components, the Erlenmeyer flask was then transferred to a shaker at 50 rpm and kept for 48 hours at room temperature. For the following step, the extract obtained was filtered through a filter paper (Wattman No.41) and then concentrated using the rotary-evaporator apparatus at 40°C. At the end, it was kept in a refrigerator at -18 °C until used. The dried extract was finally subjected to antibacterial experiments.

-The recombinant peptide synthesis

The Chinese hamster’s ovary cells, which were transfected with the pcDNA 3.1+ vector (Invitrogen, USA), were utilized for the cloning and expression of the entP gene. According to the secretional signal and the coding sequence of the recombinant EnP in the vector, the transfected cell lines had the capability of synthesizing and secreting the peptide into the medium. The transfected cell lines were then harvested, and the medium containing the recombinant peptide was collected for the antimicrobial assay.

The transfected Chinese hamster’s ovary cells were in culture in the Dulbecco Modified Eagle Medium (DMEM) (Sigma-Aldrich Co. LLC, St. Louis, USA) containing 10% of the fetal calf serum (FCS) (GIBCO Laboratories, Life Technologies, Inc., New York, USA) inactivated by heat and the combination of penicillin, streptomycin (GIBCO Laboratories, Life Technologies, Inc., New York, USA), and 2 millimole of glutamine (Biosera, Ringmer Lewes, England), cultivated in an incubator with 5% of Co2, and 85% of humidity, at 37 °C. The cells passage occurred every 2-3 days at 0.2 × 106 cell/ml of the cell concentration. After each instance of passages, the culture containing the recombinant peptide was collected from the transfected cells.

-The antimicrobial assay

For evaluation the antibacterial effectiveness of the herbs as well as the peptide, the strains of *Streptococcus mutans* (*S. mutans*) ATCC# 35668, *Streptococcus salivarius* (*S. salivarius*) ATCC# 9222 and *Streptococcus oralis* (S. oralis) ATCC# 35037 were used. The minimum inhibitory concentration (MIC) and the minimum bactericidal concentration (MBC) were determined by a broth microdilution test.

-The minimum inhibitory concentration (MIC) analysis

The experimental design involved the exposing of the selected oral pathogens in 96-well microplates for 24 hours to a dilution series of the herbal extract and essence, and EnP peptide using a microbroth dilution method. The MIC assay is normally used by microbiologists to measure the reduction of the microbial growth, usually over a fixed incubation period of about 24 hours. By conducting this test, it is meant to determine the minimum dilution of the test substance that leads to the complete growth inhibition (the undetecTable growth in the media), and the bacterial growth is measured by the change to the optical density (turbidity) of the wells in the microplate.

For the MIC analysis, 100μL of the Mueller Hinton liquid (Merk, Darmstadt, Germany) was first added to each well of the 96-well microtiter plates. Afterwards, 100μL of EnP and the highest concentration of the extract and essence (1000 µg /ml) were added to the first well. Other wells were also filled with lower concentrations up to the 10th well (1.8 µg /ml). Well number 11 just contained the medium with no bacteria, considered as the negative control, and well number 12 was considered as the positive control group that contained the medium, the bacterial suspension, and 1% dimethyl sulfoxide. Later on, 10 μL of the bacterial suspension containing about 5× 105 CFU/ml was added to each well. The plates were then incubated at 28 °C in an incubator for 24 hours. After the termination of the incubation period, the well turbidity was inspected visually and read by the Eliza device (STAT FAX 2100, Minnesota, USA) at 630 nm. The first well in the series with no sign of visible bacterial growth was considered as MIC. MIC is the antimicrobial agents of the lowest concentration that visually inhibit 99% of the growth of microorganisms.

-The minimum bactericidal concentration (MBC) evaluation

MBC was determined by culturing 5μL of the contents of the wells with no sign of bacterial growth on the nutrient agar. The plates were incubated for 24 hours at 28°C in an incubator. The least concentration that inhibited 99.9% of the colony forming of oral pathogens on the nutrient agar was considered as MBC.

The entire procedures of MIC and MBC tests were repeated for the third time, and the data were expressed in a descriptive analysis (the mean).

-The cytotoxicity assay

To determine the toxicity of EnP, the primary culture of the gingival fibroblast cells was used. The gingival fibroblast cells were cultured in DMEM (the Dulbecco’s modified eagle medium) containing 5% fetal bovine serum (FBS), 100 unit/mL of penicillin, and 100μg/mL of streptomycin sulfate. They were then incubated at 80% humidity with 5% CO2 at 37°C in an incubator, for 24 hours. Next, the cells were cultured at the different concentrations of plants and EnP for 24, 48, and 72 hours. The viability of the cells was determined by the MTT test to measure the concentration and time of the toxicity of the agent tested.

-MTT assay

After the cell passage process for the MTT evaluation, the cell count was adjusted at 3000-4000 cells for each DMEM well. The viability power was evaluated using 3-(4,5- dimethylthiazole-2) 2,5- diphenyl tetrazolium. Afterwards, 10μL of MTT (thiazolyl blue tetrazolium dye) dissolved in DMEM was added to each well and incubated for an hour in the incubator at 37°C. Six wells containing the medium with no extract were considered as the control group. After discarding the cell medium, 100 μL of dimethyl sulfoxide (DMSO) was added to the precipitated cells. Absorbance was then measured using a microplate reader in the ELIZA plate reader at 570-620 nm (STAT FAX 3200, Minnesota, USA). All concentrations and times were assessed for 3 of 96 wells using triplicate measurements. The absorbance values lower than those of the control group indicated a reduction in the cell growth. The concentration required to reduce the cell growth down to less than 50% (CC50) was determined using a chart.

-The statistical analysis

Data were analyzed using the SPSS statistical software (version 22.0, IBM, Chicago, IL, USA). To evaluate statistical significance for the study groups, the one-way analysis of variance (ANOVA) was conducted, followed by the Tukey’s HSD test for the post-hoc analysis. The entire statistical analysis was performed with the significance level set at 5%.

## Results

The MIC and MBC values for both herbal plants and EnP against the assessed bacteria have been presented in Table 1 and Table 2, respectively. According to the results obtained, the MIC and MBC of both herbal plants were greater than those of the 0.2% CHX mouthwash. *C. cyminum* was found to be of the strongest effects on *S. mutans*, inhibiting the bacterial growth and killing 62.5 μg/mL of the bacteria. The other three pathogens had comparable antibacterial effects, being twice less susceptible to the bacteriostatic and bactericidal effects of *C. cyminum* (125 μg/mL) than *S. mutans*. Besides, this herbal plant demonstrated four times weaker bactericidal effects on *S. oralis* (250 μg/mL) than *S. mutans*. The *F. angulata* extract exhibited the strongest bactericidal and bacteriostatic effects only on *S. oralis*, with its MIC and MBC value being 62.5 μg/mL. The results of the bacteriostatic effects of *F. angulata* on other pathogens were comparable (125 μg/mL).

**Table 1:**
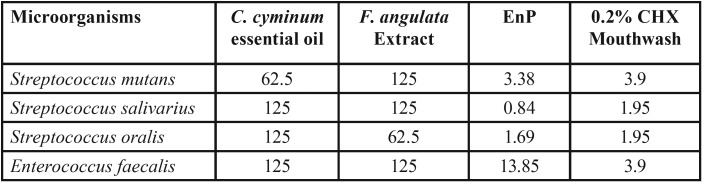
The minimum inhibitory concentration (MIC: (µg /ml)) of the *C. cyminum* essence, the *F. angulata* extract, and EnP against the bacteria involved in dental caries and endodontic infections as against 0.2% chlorhexidine (CHX) mouthwash.

**Table 2:**
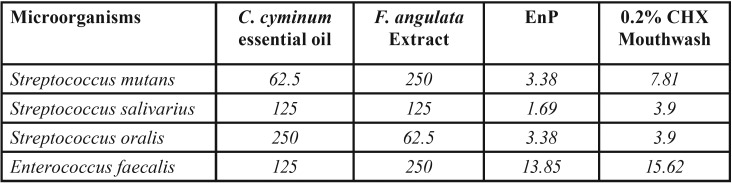
The minimum bactericidal concentration (MBC: (µg /ml)) of the *C. cyminum* essence, the *F. angulata* extract, and EnP against the bacteria involved in dental caries and endodontic infections compared with 0.2% chlorhexidine (CHX) mouthwash.

EnP displayed the MIC values of 0.84-3.38 μg/mL and the MBC values of 1.69-3.38 μg/mL against cariogenic strains, being several times more effective than the medicinal plants. However, it exerted less strong effects on *E. faecalis* than streptococci with the MIC and MBC value of 13.85 μg/mL. Moreover, it was found that EnP had stronger effects on all of the bacteria tested than 0.2% CHX, except for E. faecalis where CHX exhibited stronger bacteriostatic effects than EnP.

The results obtained from the cytotoxicity assay of EnP have been presented in Table 3. The one-way ANOVA analysis showed a statistically significant difference among the concentrations (*P*=0.0), the time of assessment (*P*=0.0), and the interaction (*P*=0.01). According to this analysis, there was no statistically significant difference between the cytotoxicity of the control group and that of Enp in the three evaluations apart from after 72 hours when the cell viability at 3.75 µg/ml concentration was significantly lower than that of the control group (*P*=0.05).

**Table 3:**
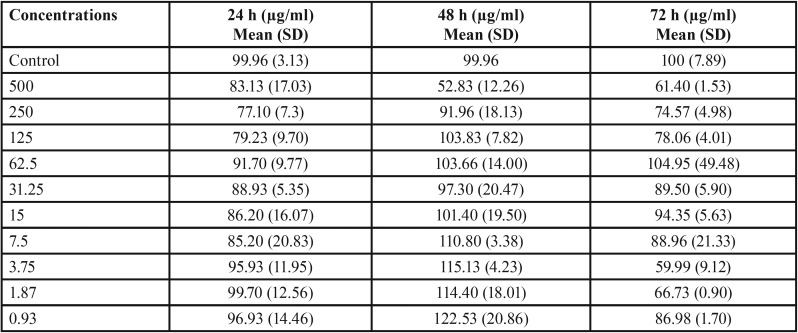
The results of the MTT analysis of EnP on human gingival fibroblasts.

Moreover, the one-way ANOVA showed that there was a significant difference between different concentrations after 48 (0.001) and 72 hours (*P*=0.039). Data from the Tukey’s HSD analysis indicated significant differences among the three cases of measurement at 125 (*P*=0.01), 3.75 (*P*=0.001), and 1.87 (*P*=0.01) µg/ml concentrations. This analysis revealed that at 125 µg/ml concentration, the cell viability increased significantly after 48 hours and decreased noticeably until the 72nd hour. Besides, there was a significant difference at 3.75 and 1.87 µg/ml concentrations for 24 and 72 hours (*P*=0.007 and *P*= 0.04, respectively), and for 48 and 72 hours (*P*= 0.001 and *P*= 0.009, respectively).

**Figure 1:**
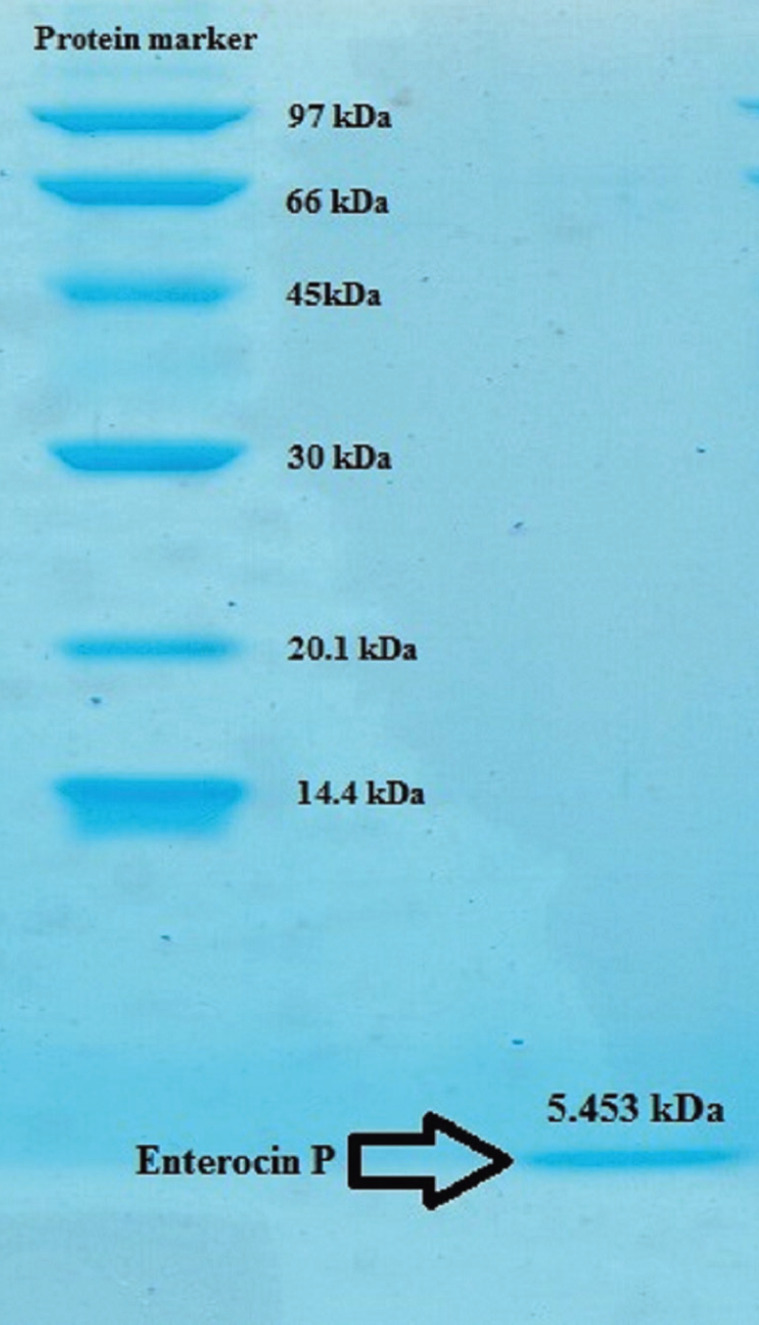
The SDS-PAGE analysis of proteins expressed in recombinant Chinese hamster ovary cells harboring Pcdna 3.1+ vector.

**Figure 2:**
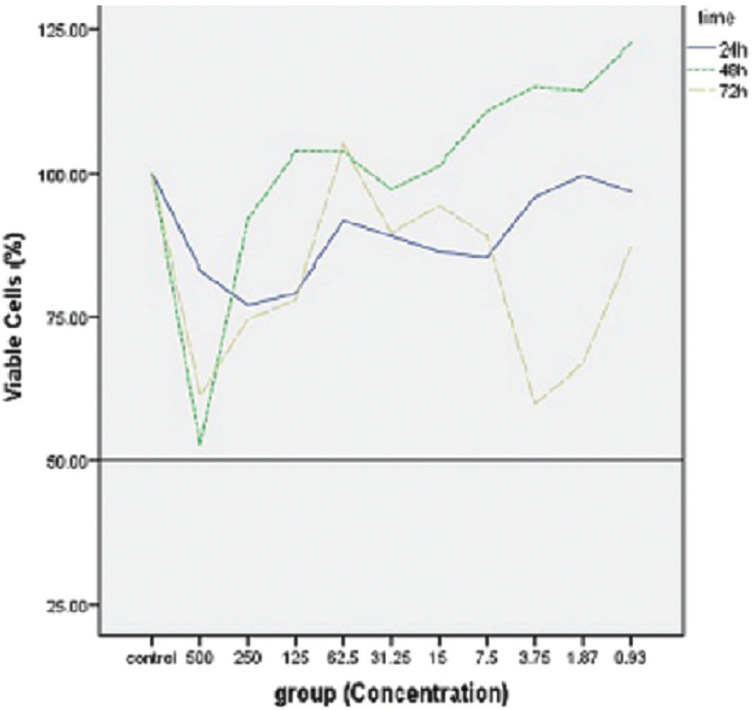
The mean cell viability (%) of gingival fibroblasts in 24, 48, and 72 hours at different concentrations of EnP compared with the control group.

The mean cell viability percentages (%) of fibroblasts in 24, 48, and 72 hours have been demonstrated in Figure 1,2, for different concentrations of EnP. As demonstrated, there has been no concentration to have led to more than 50% of the growth inhibition (IC50) of the gingival fibroblasts for the three cases of evaluation, being indicative of the biocompatibility of the EnP peptide.

## Discussion

Dental researchers are testing new therapeutic drugs to prevent or treat dental plaque-related diseases with no or the least amount of toxicity. Thus, this research was performed aimed at introducing new natural antimicrobial agents, either the same as protective human peptides or derived from plants, with the later having been recently considered more in dentistry instead of chemicals to inhibit the bacterial growth. The use of recombinant peptides with antimicrobial effects has been proposed instead of chemical agents for the protection of plants against pathogens. Chemicals could be toxic and develop resistance in microorganisms (24). As far as the researchers of this paper are concerned, this is the first report on the antimicrobial effects of the new recombinant peptide of EnP on oral pathogens.

This study was done on several Viridans Streptococci considered as the primary plaque formers that initiate dental caries. In fact, supragingival plaque is mainly composed of the gram-positive bacteria of the Viridans group, including *S. mutans*, *S. sanguinis*, *S. mitis*, *S. salivarius*, *S. oralis*, and lactobacilli (1). As against other streptococci, *S. mutans* is a highly cariogenic pathogen that can ferment carbohydrates into lactic acid, formate, ethanol, and acetate (25). Therefore, these pathogens are of considerable clinical importance in dentistry. The vast majority of the oral streptococci strains consist of commensal species, yet they can become pathogenic when the impaired host’s health, systemic immune changes, or local triggers, including oral hygiene deterioration and surgical interventions affect oral health (2).

According to the results of this study, from among all microorganisms tested, *E. faecalis* demonstrated the lowest sensitivity to the antibacterial effects of EnP and CHX mouthwash. *E. faecalis* was selected as the test organism against antimicrobial agents, since it was the most common bacterium isolated from the root canal, either alone or combined with other microorganisms (6). It is most frequently isolated in larger numbers in post-endodontic treatment cases where it is enabled to survive for a long period of time in the root canal, for possessing certain properties and virulence factors. In addition, it may develop resistance to antimicrobials and antibiotics.

To compare the antibacterial properties, the microbroth dilution method was employed in this study with the serial dilutions of a solution to determine the lowest concentration of the material that would still show antibacterial properties. Using this method, it was demonstrated that the EnP recombinant peptide was of effective properties against the tested pathogens that led to dental caries and endodontic failures, with the MIC value of 3-13 μg/ml. Therefore, it was shown for the first time that the recombinant Enp had effective antibacterial properties against oral pathogens.

Enterocins are a large group of bacteriocins, which are produced by the species of Enterococcus, including *E. faecalis*, *E. faecium*, *E. durans*, and *E. munditi*. Since many enterocins show bactericidal properties against pathogens and spoilage microorganisms present in foods, they are used as natural food preservatives to extend the half-life of foods (26).

Most bacteriocins have cationic characteristics at neutral pH, being of an hydrophobic, and amphiphilic nature (27). The amino acids, including alanine, valine, leucine, isoleucine, proline, methionine, phenylalanine, and tryptophan give proteins their hydrophobic properties, and lysine, arginine, and histidine give their cationic properties. The positively charged proteins at the cytoplasmic membrane bind to the negatively charged phospholipids of the membrane of sensitive cells and distribute throughout the membrane due to their amphipathic nature (28). After getting attached, they accumulate inside the membrane and lead to the pore formation. In the end, the cytoplasm content diffuses from the cells, resulting in bacterial death (24). Although bacteriocins are resistant to extreme pH values, temperatures, and salinity, proteolytic enzymes such as those in the pancreas (trypsin and chemotrypsin) and stomach (pepsin) can be inactivated by them. EntP has demonstrated stability at very low and high temperatures, tolerance to being exposed to pH between 2 and 11, and tolerance to repeated freeze-thawing cycles without losing its antimicrobial property (29). It has been demonstrated that EnP is generally active at acidic and neutral pH; this property justifies its application in the acidic environment of the oral cavity produced cariogenic bacteria.

Since the generation of EnP by the bacteria as a production host may carry antibiotic-resistant genes and/or genes encoding potential virulence factors, it seems it would not be an ideal production host (30). Hence, many efforts have been made to produce functional EntP in hosts other than the bacteria such as yeasts. However, in this study the recombinant form of EnP was produced by transfecting the ovarian cells of Chinese hamsters with the bacteria containing the vector.

The results of the present study demonstrated that EnP had weaker bacteriostatic and bactericidal properties against *E. faecalis* than other sterptococci. As already described, the combination of bacteriocins with other substances such as EDTA or some organic acids can yield good results against resistant and Gram-negative bacteria (26), to be investigated in future studies.

Essential plant oils are the potential sources of new antimicrobial compounds, being especially active against bacterial pathogens due to having hydrophobicity properties. Therefore, they can degrade the lipids of the bacterial cell wall and mitochondria, and subsequently destroy bacterial structures (23). Since the major components of both plants evaluated in the present study are fat soluble, the presence of ethanol or methanol as solvents during the extraction process can extract more metabolites from the plants. Besides, although water can be used as a solvent, the alcohol extracts of the plants generate more powerful inhibitory properties than the aqueous extract (2). Hence, better results can be obtained from the good solubility of active components in the organic solvent. As a result, 80% ethanol was used in this study during the extraction process, evaporated after the process was over.

According to Rios and Recio (31), and Gibbons (32), a promising plant extract must have MIC values of lower than 100 μg/mL, while pure compounds must possess MIC values of lower than 10 μg/mL. In a study on anti-staphylococcal plant-derived natural products done by Gibbons *et al*. (2004) (33), they concluded that antibacterial compounds with the MIC values of greater than 100 μg/mL were poorly active; in reality, an MIC value of less than 10 μg/mL and ideally less than 2 μg/mL is considered to be favored by pharmacologists. Based on the previous statements, although both plants demonstrated antibacterial effects in the present study, the promising results against pathogens with MIC and MBC value equaling 62.5 µg/ml were shown to be for the *C. cyminum* essential oil on just *S. mutans*, and the *F. angulata* extract on just *S. oralis*. Besides, the antibacterial effects of both plants did not reach that of EnP peptide, where they presented the MIC and MBC values of below 3.5 μg/mL against the three streptococci. It is worth mentioning that the potency of the extract was lower than that of the 0.2% CHX mouthwash, yet this is of less significance than the safety of the herbs (18-23). Since several studies have confirmed the safety and biocompatibility of the herbs, they can be applied on a long-term basis to human subjects.

According to a study by Derakhshan *et al.* (14), the essential oil of Cumin seeds demonstrated significant antibacterial properties against *K. pneumoniae* *in vitro*. In addition, the strains exposed to the sub-MIC levels of the essential oil exhibited an impaired ability to form a biofilm compared to the control group. The results of the study by Abbaszadegan *et al.* (23) on the antibacterial properties of Cumin against endodontic pathogens and its biocompatibility on fibroblasts were partially in agreement with the present research. The authors evaluated the antimicrobial and cytotoxicity properties of the Cumin essential oil and compared it with chlorhexidine (CHX) and co-trimoxazole on planktonic and biofilm forms of the bacteria isolated from teeth with persistent endodontic infections. They reported that Cumin exhibited strong antimicrobial properties against the microbial flora of the teeth and co-trimoxazole, being biocompatible with L929 mouse fibroblasts. The MIC and MBC values of Cumin were 185.91 and 175 μg/mL, respectively in their study, while those values were 62.5-125 μg/mL in the current one. Besides, Abbaszadegan *et al.* (23) employed the L929 cell line of mouse fibroblasts that is a well-characterized cell model previously used to assess the cytotoxic effects of dental materials. However, human gingival fibroblast cells were employed in the present study. Due to the methodological differences, it is difficult to directly compare the results of their study with those of the current research. In line with the findings of the present study, a research on Tunisian Cumin reported that the MIC values of the Cumin essential oil were about 78-150 μg/mL against a group of Gram-positive and Gram-negative microorganisms, including *E. faecalis* (34).

The data from the current study revealed the highest bacteriostatic and bactericidal properties of *F. angulata* against *S. oralis* (62.5 μg/mL). The bacteriostatic properties of *F. angulata* against *S. salivarius*, *S. mutans*, and *E. faecalis* strains were comparable (MIC= 125 μg/mL), being about half of that value against *S. oralis* (62.5 μg/mL). This medicinal plant presented the weakest bactericidal properties against *S. mutans* and *E. faecalis* (MBC= 250 μg/mL), being four times weaker than *S. orali*s (MBC= 62.5 μg/mL) (Table 1). There were some controversies over the antimicrobial effects of *F. angulata* in the literature. In a study by Taran *et al.* (35), the researchers reported some antimicrobial effects for the essential oils obtained from the aerial parts and seeds of F. angulata against the bacteria and fungi tested. In contrast, in the study by Hosseine *et al.* (11), the essential oils and extracts of Ferulago angulata exhibited no considerable biological properties. It is not possible to find any contradiction between the results presented by the aforementioned authors and the present ones, considering that this plant was tested on several oral pathogens.

These controversies and trivial differences may be connected with the ingredients of the plant. The composition of herbal essential oils can be variable depending on several factors, such as the geographic region, harvest time, extraction method and type of culture. However, this diversity in the composition can be regarded as a pharmaceutical opportunity so that different therapeutic usages of the same plant species grown in different regions will become possible. In addition, the differences in antimicrobial susceptibility tests can be explained by the variations in the methodologies, as several factors (including the inoculum amount, medium composition, pH, and incubation) can influence the interaction between microorganisms and antimicrobial agents, affecting the values obtained for MIC and MBC.

Relying on the previous studies that reported the safety of the *F. angulata* extract (18-21) and Cumin (22,23) on normal cells, this study did not assess the cytotoxicity of those plants. Khafagi *et al.* (22) evaluated the cytotoxicity of seven herbal essential oils against shrimp larvae (Artemia Salina), using a method commonly applied as a cytotoxicity assay in pharmacology. The authors concluded that the LC50 of Cumin was over 1000 µl/ml, being about 200 times more than that of Clove and Rosemary essential oils. This concentration is by 10 times more than the effective dose that may be used against pathogens generating dental caries and endodontic failures (62.5 µg /ml). In agreement with the current study, Shahneh *et al.* (20,21) did not allude to any inhibitory effects of the F. angulata extract on the proliferation of the normal HUVEC cell line (human umbilical vein endothelial cells) after 24 and 48 hours of incubation.

Due to the low cost and availability of herbs in different parts of the country, the use of these medicinal plants in preventing and treating oral conditions can have occasional benefits for rural communities or people in poor socioeconomic conditions. Although medicinal plants can be good alternatives to standard oral antimicrobials, there are several drawbacks to the routine usage in the general clinical practice. First of all, standardization is difficult as the chemical constituents of plant-derived products often vary. Besides, the contents of the active constituents of plants seem to be variable. As already stated, the plant family and the country of origin play a significant role in the antimicrobial properties of herbals (36).

According to the literature, chlorhexidin (CHX) exhibited the strongest antimicrobial effects on oral pathogens, so it can be considered as the quality standard for the dental plaque control. Therefore, other agents should be compared with CHX in terms of antibacterial efficacy. However, the present significant cytotoxicity on human fibroblast cells (1) resulted in searching for the better alternatives of natural origins (6,37). The results obtained from the present study revealed comparable or even more effective antibacterial properties for EnP than CHX against oral pathogens, and in the meantime no cytotoxicity was shown on fibroblast cells for EnP. As a result, this peptide can be used as a safe alternative for CHX and other chemicals in dental fields for the prevention and management of oral bacteria-induced diseases.

One of the limitations of this study was that the killing time of the tested components was not evaluated. Time-kill tests provide valuable information about the time during which antimicrobial agents can eradicate pathogens. To obtain more accurate results, relevant studies are suggested to investigate the killing time of antimicrobial agents.

In this study aimed at comparing two medicinal plants with a recombinant peptide of EnP, the authors concluded that the EnP with better antibacterial properties can act better against oral pathogens, being considered as a promising alternative for chemicals in oral disease management, compared with both herbal plants and CHX. It was also demonstrated that the antimicrobial effects of EnP against *E. faecalis* and three caries-inducing streptococci were several times stronger than herbal plants tested in this study. Additional clinical and laboratory studies should be performed to evaluate the beneficial use of this peptide as an additive to intracanal irrigant, or any other preventive materials. Although herbal plants are frequently used in medical fields, especially by traditional peoples, further studies are necessary to evaluate the possible toxicity effects of these components on animals or human subjects as well as their application in the medical system, before making any assertion.

## Conclusions

Considering the remarkable antibacterial effects of the recombinant peptide of EnP on *E. faecalis* and caries-inducing streptococci compared with herbals, and taking into account the biocompatibility of this peptide, it can be used as a substitute for chemicals in managing oral pathogens. The involvement of EnP as a new approach in dentistry into the intra-canal medicaments, root canal sealers, and also in irrigating solutions can eradicate *E. faecalis* from the root canal, thereby increasing the success rate of root canal treatments. Furthermore, it can be added to oral hygiene products and dental materials to prevent dental caries and maintain oral health. However, to achieve these goals, further *in vivo* studies are required to apply it more effectively.
